# Ant-following behavior is correlated with plumage traits in African understory birds

**DOI:** 10.1007/s00114-024-01927-3

**Published:** 2024-07-30

**Authors:** Matthias Waltert, Janina Klug, Francis Njie Motombi, Benjamin Cejp, Kadiri Serge Bobo, Mahmood Soofi, Marcell K. Peters

**Affiliations:** 1 Department of Conservation Biology, J.F. Blumenbach Institute of Zoology and Anthropology, Georg-August-University of Göttingen, Bürgerstrasse 50, 37073 Göttingen, Germany; 2 Environment and Geology (HLNUG), Hessian Agency for Nature Conservation, State Institute for the Protection of Birds, Europastraße 10, 35394 Giessen, Germany; 3 Mount Cameroon National Park, Buea, Cameroon; 4 Evolutionary Ecology, Institute of Organismic and Molecular Evolution, Johannes Gutenberg-University Mainz, Mainz, Germany; 5 Department of Forestry, University of Dschang, P.O. Box 222, Dschang, Cameroon; 6 CSIRO Land and Water, PMB 44, Winnellie, Darwin, Australia; 7 Geography Department, Humboldt-University Berlin, Unter den Linden 6, 10099 Berlin, Germany; 8 Department of Animal Ecology and Tropical Biology, Biocenter – Am Hubland, Julius-Maximilians-Universität Würzburg, Würzburg, Germany

**Keywords:** Afrotropical, Driver ants, Aggression, Dominance, Plumage, Morphology

## Abstract

Ant-following behavior is a common phenomenon in birds of Neotropical and Afrotropical rainforests but yet little is known from Central Africa. We here report on the phenomenon in lowland rainforest in Cameroon, quantifying the strength of the interaction of different ant-following bird species with driver ants and test the hypothesis that higher levels of specialization in ant-following behavior are associated with dominance or aggression-dependent plumage and other morphological traits. Flock size varied between 1 and 11 individuals with a mean size of 5.34 ± 2.68 (mean ± SD) individuals occurring at the same time. The maximum number of species present during one raid observed was ten, whereas the minimum number was four with an overall species richness of 6.89 ± 2.1 species. The 21 attending bird species strongly varied in the degree of ant-following behavior. In an interspecific comparison, plumage traits such as the presence of a colored crown, eyespots, and bare skin around the eye, in combination with metatarsus length and weight, were significantly correlated with ant-following behavior. These results suggest that—in size and identity of species—ant-following bird assemblages in Central Africa are similar to those reported from East Africa. They also suggest that ant following favors the selection of traits that signal dominance in interactions between individuals struggling for valuable food resources in the forest understory.

**Supplementary Information:**

The online version contains supplementary material available at 10.1007/s00114-024-01927-3.

## Introduction

Ant-following birds engage in the exploitation of swarm-raiding army ants, which are keystone predators in tropical forests, foraging on cryptic arthropods and vertebrates as they escape from raiding army ants (Brady 2003; Kronauer 2009). This behavior is a well-documented phenomenon for Central and South America (basic knowledge: Willis and Oniki 1978; O’Donnell et al. 2012; questions on cognitive bird adaptations: Logan et al. 2011) and has also been described for Afrotropical birds (Willis and Oniki 1978). Research on ant-following birds over the last few decades has nevertheless mainly focused on the Neotropics, and there are only a few recent systematic studies on the association between birds and ants of the African continent (Peters et al. 2008; Peters and Okalo 2009; Peters 2010; Craig 2022). In Africa, army ants associated with birds belong to the subfamily Dorylinae, subgenus *Anomma*, also referred to as driver ants (Chapin 1932; Willis and Oniki 1978; Brady 2003). Similar to the Neotropical *Eciton burchellii*, these ants conduct surface swarm raids spanning an area of several square meters. During these raids, the ants flush invertebrates from the upper soil and leaf litter, which thus become easily accessible to insectivorous birds. The army ant syndrome also occurs in the Asian Dorylinae genus *Aenictus*, but no association with birds has been observed yet, since most *Aenictus* species are hypogaeic (living within the soil) in their activities (Gotwald 1995; Brady 2003).

Compared to the Neotropical army ants, African driver ants conduct raids which appear to be less predictable and may be triggered by both food depletion near the nest as well as by attacks from other ant species or even mammalian predation (Willis 1983, 1985, 1986; Gotwald 1995; Sanz et al. 2010). In Kakamega Forest, Kenya, Peters et al. (2008) identified 56 species of birds attending raids of the driver ants *Dorylus wilverthi* and *D. molestus*. Of these, five species were quantitatively acknowledged as specialized ant-followers, mainly belonging to the thrushes Turdidae (1 sp.), thrush-like Muscicapidae (3 spp.), and bulbuls (1 sp.). A recent review suggests that among 958 bird species occurring in driver ant habitats, 168 bird species had been mentioned in the literature to have been recorded with ants, with 116 being occasional and 52 regular ant-followers (Craig 2022). Besides the works by Peters et al. (2008), systematic studies regarding the relative strength of the association of species listed are, however, still scarce. Furthermore, the association between ant-following behavior and morphological and behavioral traits, which are known from Neotropical species, remain principally untested (Craig 2022).

In the Neotropics, many obligate ant-following bird species possess ornaments such as bare, colored spots around the eye (several Thamnophilidae), bright supercilia (e.g., Rufous-capped warbler *Basileuterus rufifrons*), colored crowns (e.g., blue-crowned motmot *Momotus momota*), or bright spots on the face (e.g., white-eared ground sparrow *Melozone leucotis*), but also unornamented species have been identified as obligate ant-followers (Willson 2004; O’Donnell et al. 2010).

In numerous bird species, size and color of plumage ornaments and other morphological characteristics play a role in social behavior, both intrasexually in competition between males and intersexually through mate choice (Senar and Quesada 2006; Mason and Bowie 2020). But plumage traits have been found to signal aggression and dominance in competitive contexts beyond sexual selection (Swaddle and Witter 1995; Tobias et al. 2012). Avian ornaments consist generally of carotenoid-based, melanin-based, or structural (blue) colors (Jawor and Breitwisch 2003), as well as of unpigmented feathers (e.g., Galvan 2010). If more than one ornament is present in a species, not all do necessarily play a role in intraspecific competition (Young et al. 2016), but can be redundant (Jawor and Breitwisch 2004) or have different meanings (Badyaev and Hill 2000). In Thamnophilidae, signals evolving from social selection/for intraspecific communication have been shown to function in an interspecific context as well (Tobias and Seddon 2009).

Not all ant-following species depend on arthropods flushed by ants in the same manner: the variety ranges from occasional ant-followers that only prey on arthropods flushed when the army ant raid crosses their territory, to obligate ant-followers that meet most of their dietary needs this way (Willson 2004; Chaves-Campos 2011). Antbirds are especially interesting for studying interspecific competition, as during ant raids, birds from different species and with different degrees of specialization gather at a small, temporarily available food source (Martinez et al. 2021). Even though different foraging strategies may lower direct competition (Willis and Oniki 1978), direct encounters between competing species are unavoidable (O’Donnell et al. 2012). In some species, strong interspecific competition and aggression may even lead to a complete mutual avoidance at raids (O’Donnell 2017). Body mass seems to correlate with interspecific dominance in ant-following birds (Willson 2004; Willis and Oniki 1978), but the role of plumage traits in this context has yet to be studied (Mason and Bowie 2020).

Among the species Peters et al. (2008) found to be associated with army ants in Kenya, there are species with plumage ornaments such as bright supercilia (e.g., grey-winged robin *Sheppardia polioptera*, brown-chested alethe *Chamaetylas poliocephala*) or bare, colored patches around the eyes (e.g., red-tailed bristlebill *Bleda syndactyla*), while also relatively more dull colored species attended ant swarms (e.g., yellow-whiskered greenbul *Andropadus latirostris*, brown illadopsis *Illadopsis fulvescens*). Among the most specialized species, many are terrestrial, belong to the families Turdidae, Pycnonotidae, or Timaliidae (Waltert et al. 2005), and have long tarsometatarsi, presumably as an adaptation to this lifestyle. Nevertheless, the relationship between morphological traits and the degree of ant-following behavior remained untested.

We here describe for the first time the ant and bird association for a West African forest and examine the relationship between the degree of ant-following and plumage traits which have been hypothesized to signal dominance. We first assessed the relative abundances of ant-following birds against their frequency in ant raids, to estimate the strength of each species’ association with ant swarms. In an interspecific comparison, we then modeled the strength of this association against plumage and other morphological traits. We hypothesized that the strength of the association between ant-following birds and ants is tied to a species’ morphological characteristics associated with terrestrial foraging, as well as with aggressive behavior, indicating intense competition for important resources.

## Methods

### Study area and field work

We conducted our surveys between April and June 2016 in Korup National Park (KNP; N5° 16′ 46.139″ E9° 3′ 28.645″) in the Southwest of Cameroon (West Africa). KNP covers an area of 1260 km^2^ and shares a border with Nigeria in the Northwest. The park belongs to the “Guinean Forests of West Africa,” with at least 551 mammal and 514 bird species (Oates et al. 2002). Korup itself holds more than 400 bird species (Rodewald et al. 1994; Green and Rodewald 1996). At least 15 species of birds known to occur in KNP have been observed following swarms of ants or joining mixed-species flocks around the swarms (Rodewald et al. 1994; Keith et al. 1992).

We selected a ca. 13 km^2^ study site in the southern sector of KNP in proximity to the so-called Chimpanzee Camp. There, 12 transects with north–south-orientation were already established and used in studies on duikers (see, e.g., Viquerat et al. 2012); we were using 10 out of these 12 transects for our study and conducted point counts every 200 m along each transect. We surveyed each of these points three times, representing three distinct time frames of the day: (1) the early morning, (2) closer to noon, and (3) between afternoon and evening. We started at the transect depending on its distance to the camp. Earliest start of a morning survey session was at 06:44 a.m. The midday session started between 09:30 and 10:00 a.m. For the afternoon session, we did not start before 02:40 p.m. to avoid the great heat during midday, when most birds usually show less activity. The point counts lasted for 10 min (following Jankowski et al. 2009); all birds appearing and or singing within a 100 m radius around the point were reported with distance and identified on species level.

Between 11th of April and 15th of June, a total of 36.61 km transect lines were walked, and 168 point counts performed on 10 transects and 69 points. To identify species attending army ant raids in the study site and determine species composition of flocks, observations of bird flocks associated with army ant raids were performed upon occasion over the whole study period, but non-systematic in regard to location, resulting in a total of nine flock observations. From an inconspicuous position, so as not to disturb the animals, we located and tracked raids of *Dorylus* spp*.* and documented bird species attending them for a maximum of 1 h. The point of observation was located at the front of swarm raids to ensure the documentation of the chronological development of bird flocks. As a first step of the observation, we noted down all bird species present and the number of each species at the moment of arrival, along with the time of arrival (Coates-Estrada and Estrada 1989; O’Donnell et al. 2010). In order to categorize birds as army ant raid attendants, we aimed for observations of feeding on flushed arthropods, but in our main analysis, we included also species which were not observed feeding at swarms (but see Fig. 9 of Supplementary Material C). Following Peters et al. (2008), we determined species richness by counting the number of bird species within 60 min of observation. Since birds from the same species were individually indistinguishable, birds eventually leaving and returning again could not be distinguished from newly arriving individuals at the swarm. Consequently, we used the maximum number of individuals of one species present during the observation period when estimating the maximum size of bird flocks (Coates-Estrada and Estrada 1989; Peters et al. 2008).

### Determination of species

Field work and bird species identification were conducted by J.K. and F.N.M. We identified birds via visual and acoustic cues in the field, whereas ant identification at species level was performed in the laboratory. Therefore, we took 21 samples of ant specimens, at least one large soldier from each colony found, and kept them in 95% ethanol solution for a detailed analysis. For ant identification, we used identification keys from the literature (Gotwald 1982; Borowiec 2016).

### Data analysis

To estimate density for each species (ind./km^2^) within the study area, we evaluated data by using DISTANCE (Thomas et al. 2006). Bird observations were entered as clusters with perpendicular distance (single observer approach) to the bird individual or the center of a bird group. Observations beyond 100 m distance were discarded already in the field; right truncation was done at the level of species, and “half-normal keys” were used to model detection probability.

### Statistical analysis

Following O’Donnell et al. (2010), we estimated for each species its attendance at army ant raids by calculating for each species (ra-I) the proportion of raids each species attended and also (ra-II) the proportion of individuals each species contributed to all raids (Table 2, Supplementary material C). A two-way likelihood ratio test was used to assess the goodness of fit of the relationship between species’ density and occurrence at swarms of army ants. To calculate each species’ specialization towards army ant raids, we related each species’ proportion at army ant raids to their estimated density in the study site using the point count data (Peters et al. 2008; O’Donnell et al. 2010). In the resulting model, data points for bird species which attend army ant raids at a proportion which reflects their overall density in the field will be close to the regression line. In contrast, data points of birds which occur more (or less) often at swarm raids than predicted by their overall density will be indicated by high deviation from the model predictions, being outside the 95% confidence interval (CI) of the model. As a quantitative measure for specialization (i.e., specialization index), we used the residuals of this model. Accordingly, specialized army ant raid-attending bird species were identified by residuals higher than the upper 95% confidence interval (CI) of the likelihood ratio test and graphical interpretation (Peters et al. 2008; O’Donnell et al. 2010). For our results, we decided to use data from the proportion of raids attended (ra-I), but we also report (Table 2, Supplementary material C) the proportion of individuals at raids (ra-II).

### Morphological and plumage traits

For all the bird species recorded, we noted whether they showed obvious plumage traits such as a marked crown, the occurrence of an eyespot, a bare eyepatch, and a marked tail or chest (presence/absence). We also combined the occurrences of these morphological characteristics in a single binary variable *plumage traits*: out of 20 analyzed species, 11 species showed one of these traits, while 9 did not. We also gathered the metric covariates *metatarsus length*, *beak size*, and *weight* (all continuous variables) for each species from mist netting data we collected in previous studies (unpublished data). We tested if the specialization index (residuals of the regression) based on each bird species’ proportion at swarm raids (ra-I, see above) can be predicted by morphological features of the birds: We first checked multi-collinearity of the a priori hypothesized predictor variables by calculating variance inflation factor (VIF) and excluded predictor variables if the VIF > 3 using “vif” function of R “usdm” package (Zuur et al. 2010). We then standardized all the predictor variables before running models (Gelman 2008). We applied a generalized linear model (GLM) with Gaussian link function and ranked the models based on their delta (*Δ*) values by multi-model inference using the “MuMIn” package (Barton 2020) in R programming language (R Core Team 2016). Finally, we used Akaike’s information criterion corrected for small sample size (AICc) to select the best models with *Δ* < 2 (Burnham and Anderson 2002). We considered variables as significant if their confidence intervals did not include zero.

## Results

During the 168 10-min point counts, 2265 individuals were detected, belonging to 59 different bird species. Sixty individuals could not be determined at species level (2.6%). While walking transects, we were able to detect 2144 individuals from 62 bird species with 42 unidentified individuals (2.9%). Seven species were exclusively recorded when walking on line transects and four exclusively during point counts. Of the three collective and aboveground foraging *Dorylus* species known to occur in KNP, two ant species were found taking part in the association with birds during the field period: *D. mayri* Santschi, 1912 and *D. sjoestedti* (formerly *D. nigricans sjoestedti*, Emery, 1899) as identified by samples taken from worker ants of each ant swarm encountered within the study site.

### Birds at army ant raids

During field work, 22 swarms of army ants were observed, but only 9 of them were in association with birds. Within the 9 bird aggregations observed at army ant raids, a total of 88 individual birds belonging to 21 different species were detected. Aggregation size varied between 1 and 11 individuals with a mean size of 5.34 ± 2.68 individuals occurring at the same time (mean ± SD). The maximum number of species present during one raid observed was ten, whereas the minimum number was four with an overall species richness of 6.89 ± 2.1 species (mean ± SD). Bird species accumulation curves (Supplementary Material A) showed no saturation. Using the abundance-based coverage estimator (ACE Mean), we estimated a maximum number of 32 species, so that the 21 observed species represent approximately 68% of the estimated total number of ant-following birds in the area.

Of the 21 bird species detected at ant raids, there were one species each belonging to the orders Cuculiformes and Columbiformes, while the remaining 19 were Passeriformes. The family Cuculidae (within Cuculiformes) was represented by the chattering yellowbill, *Ceuthmochares aureus*, and the family of Columbidae (within Cuculiformes) was represented by the blue-headed wood dove, *Turtur brehmeri*. The 19 remaining Passeriformes represented individuals of nine families with Pycnonotidae having contributed six species, Muscicapidae, Turdidae, Pellorneidae, and Macrosphenidae, two species and the remaining single species (Table 1).

**Table 1:** Passerine bird species observed in proximity to ant raids in Korup National Park, Cameroon. Nomenclature follows Borrow and Demey (2014)

Family	Vernacular name	Scientific name	Author
Pycnonotidae	Red-tailed bristlebill	*Bleda syndactylus*	(Swainson, 1837)
	Lesser bristlebill	*Bleda notatus*	(Cassin, 1857)
	Red-tailed greenbul	*Criniger calurus*	(Cassin, 1857)
	Eastern-bearded greenbul	*Criniger chloronotus*	(Cassin, 1860)
	Yellow-whiskered greenbul	*Eurillas latirostris*	(Strickland, 1844)
	Little greenbul	*Eurillas virens*	(Cassin, 1858)
Muscicapidae	Forest robin	*Stiphrornis erythrothorax*	Hartlaub, 1855
	Fire-crested alethe	*Alethe castanea*	(Cassin, 1856)
	Brown-chested alethe	*Chamaetylas poliocephala*	(Bonaparte, 1850)
Turdidae	White-tailed ant thrush	*Neocossyphus poensis*	(Strickland, 1844)
	Rufous flycatcher thrush	*Stizorhina fraseri*	(Strickland, 1844)
Timaliidae	Pale-breasted illadopsis	*Illadopsis rufipennis*	(Sharpe, 1872)
	Brown illadopsis	*Illadopsis fulvescens*	(Cassin, 1859)
Macrosphenidae	Grey longbill	*Macrosphenus concolor*	(Hartlaub, 1857)
	Yellow longbill	*Macrosphenus flavicans*	Cassin, 1859
Monarchidae	Red-bellied paradise flycatcher	*Terpsiphone rufiventer*	(Swainson, 1837)
Sylviidae	Green hylia	*Hylia prasina*	(Cassin, 1855)
Nectariniidae	Olive sunbird	*Cyanomitra olivacea*	(Smith, 1840)
Platysteiridae	Yellow-bellied wattle eye	*Dyaphorophyia ansorgei*	Hartert, 1905

### Raid attendance of birds

**Fig. 1:**
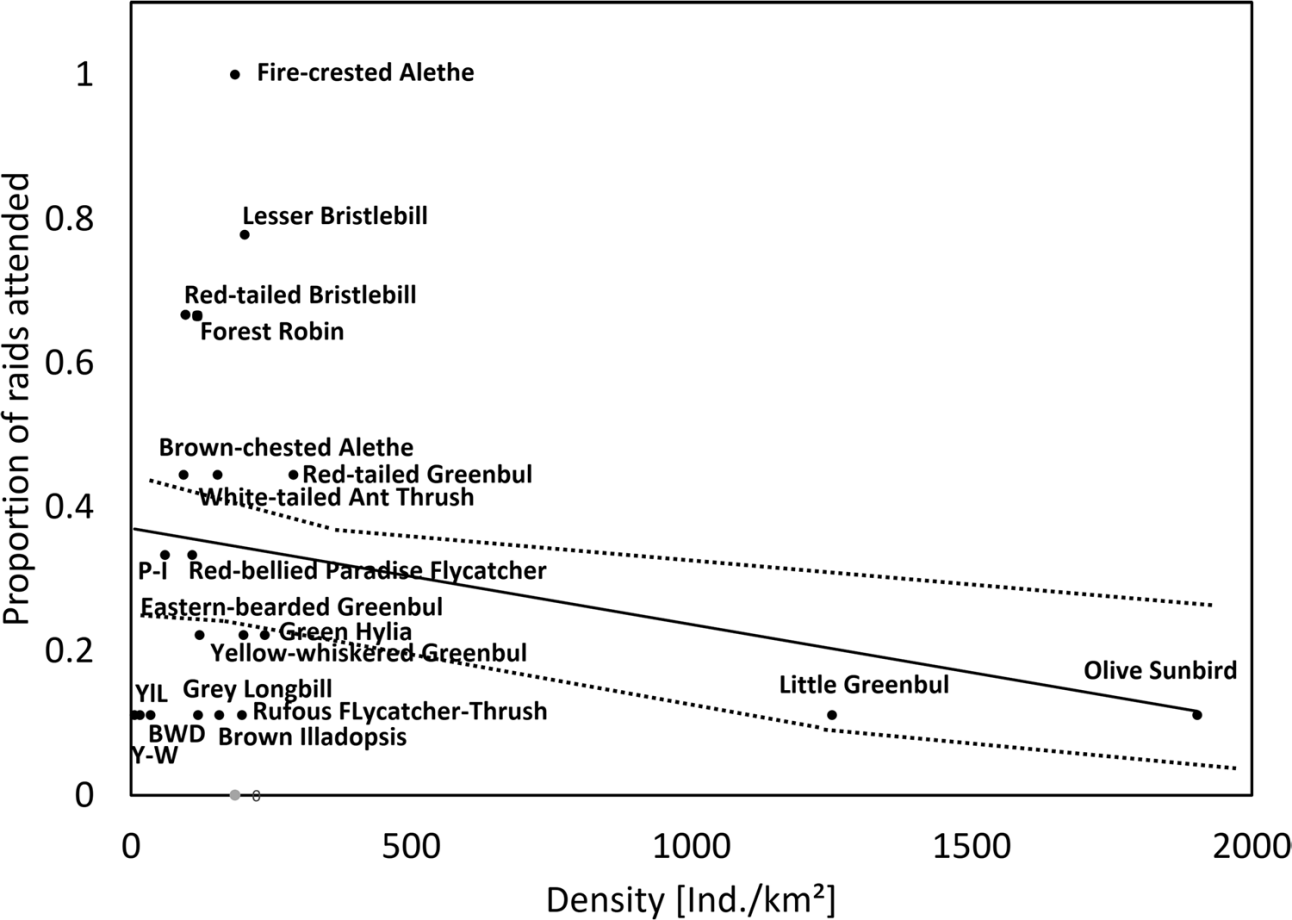
Interspecific comparison of bird species’ proportions of raids attended (ra-I, Table 2) against their density from point counts in Korup National Park, Cameroon. Species above the 95% confidence interval line (upper dashed line) are identified as specialized ant-followers. Density is slightly negatively related (*p* < 0.1, black solid line) to raid attendance ra-I, and this relationship was strongly influenced by the values of little greenbul and olive sunbird. Abbreviations: BWD, blue-headed wood dove; P-I, pale-breasted illadopsis; Y-W, yellow-bellied wattle eye; YlL, yellow longbill

The raid attendance of bird species by proportion of raids attended (ra-I, proportional) varied between 11.1% (e.g., yellowbill, yellow longbill, olive sunbird, or little greenbul) and 100% in case of the fire-crested alethe (Table 2, Fig. 1), meaning that this species was part of all aggregations observed. Raid attendance by proportion of numbers of individuals observed at raids (ra-II, proportional) varied between 1.14% of all individuals at raids (e.g., yellowbill, rufous flycatcher-thrush, and the two abundant species olive sunbird and little greenbul) and 23.86%, i.e., the fire-crested alethe, which contributed the highest portion of all individuals (Table 2). The fire-crested alethe was therefore by far the most frequent and most constant ant-follower at the study site.

**Table 2:** Bird-ant association data for species observed at driver ants in Korup National Park, Cameroon: number of raids attended and observed numbers of individuals, as well as their respective proportions (raids attended from all raids observed [ra-I] and individuals attended from all individuals [ra-II])

Species	# raids attended	% raids (ra-I)	# individuals at raids	% individuals (ra-II)
Fire-crested alethe	9	100.0	21	23.86
Lesser bristlebill	7	77.8	11	12.50
Red-tailed bristlebill	6	66.7	9	10.23
Forest robin	6	66.7	7	7.95
Red-tailed greenbul	5	55.6	6	6.82
Brown-chested alethe	4	44.4	6	6.82
White-tailed ant thrush	4	44.4	6	6.82
Red-bellied paradise flycatcher	3	33.3	4	4.55
Pale-breasted illadopsis	3	33.3	3	3.41
Eastern-bearded greenbul	2	22.2	2	2.27
Green hylia	2	22.2	2	2.27
Yellow-whiskered greenbul	2	22.2	2	2.27
Blue-headed wood dove	1	11.1	1	1.14
Brown illadopsis	1	11.1	1	1.14
Grey longbill	1	11.1	1	1.14
Little greenbul	1	11.1	1	1.14
Olive sunbird	1	11.1	1	1.14
Rufous flycatcher thrush	1	11.1	1	1.14
Yellow longbill	1	11.1	1	1.14
Yellow-bellied wattle eye	1	11.1	1	1.14
Yellowbill	1	11.1	1	1.14

### Specialization in regard to ant following

**Fig. 2:**
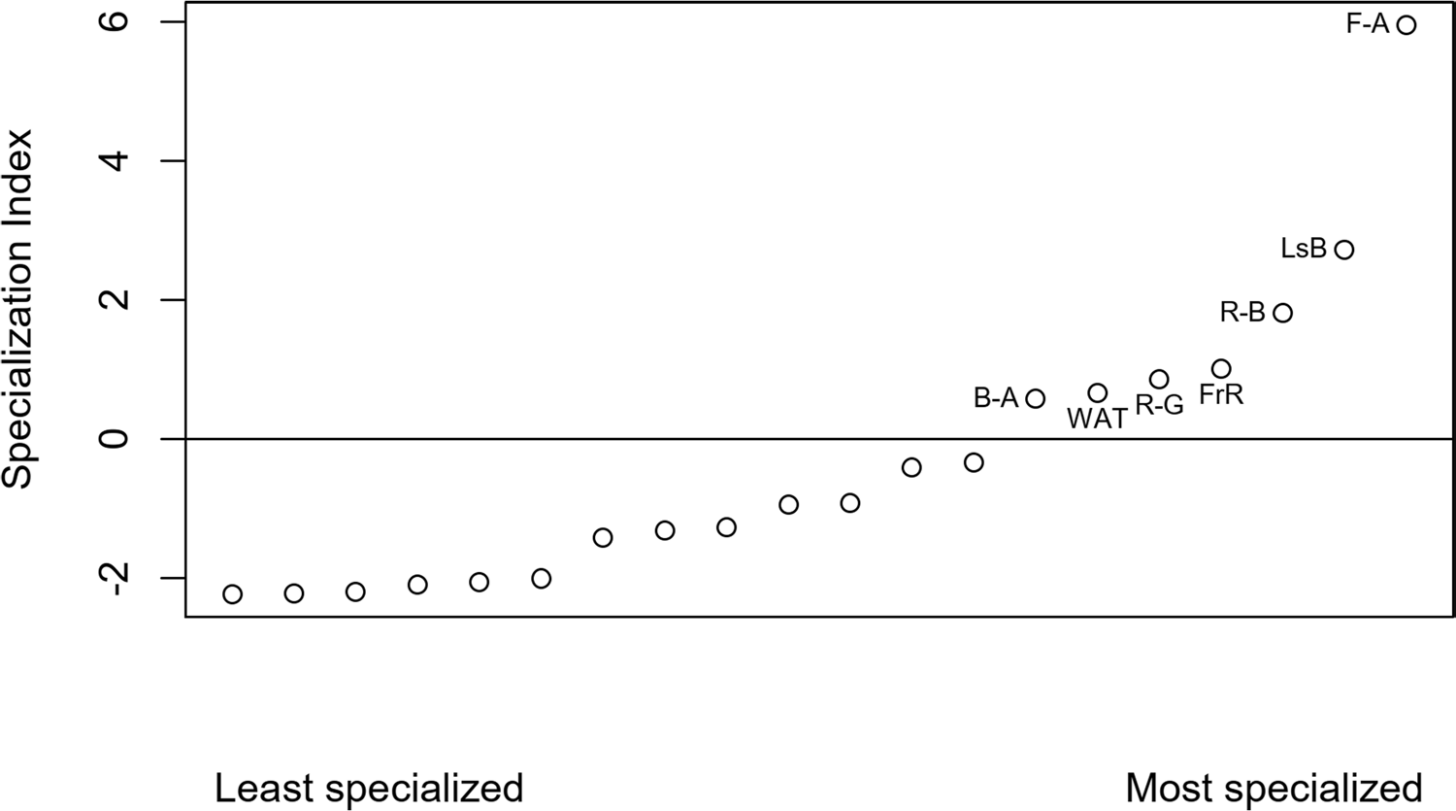
Driver ant-following indices of different bird species in lowland forest of Korup National Park, Cameroon, by proportion (%) of raids attended in ordered sequence. Labels for specialized species: B-A, brown-chested alethe; F-A, fire-crested alethe; FrR, forest robin; LsB, lesser bristlebill; R-B, red-tailed bristlebill; R-G, red-tailed greenbul; W-A, white-tailed ant thrush

**Fig. 3:**
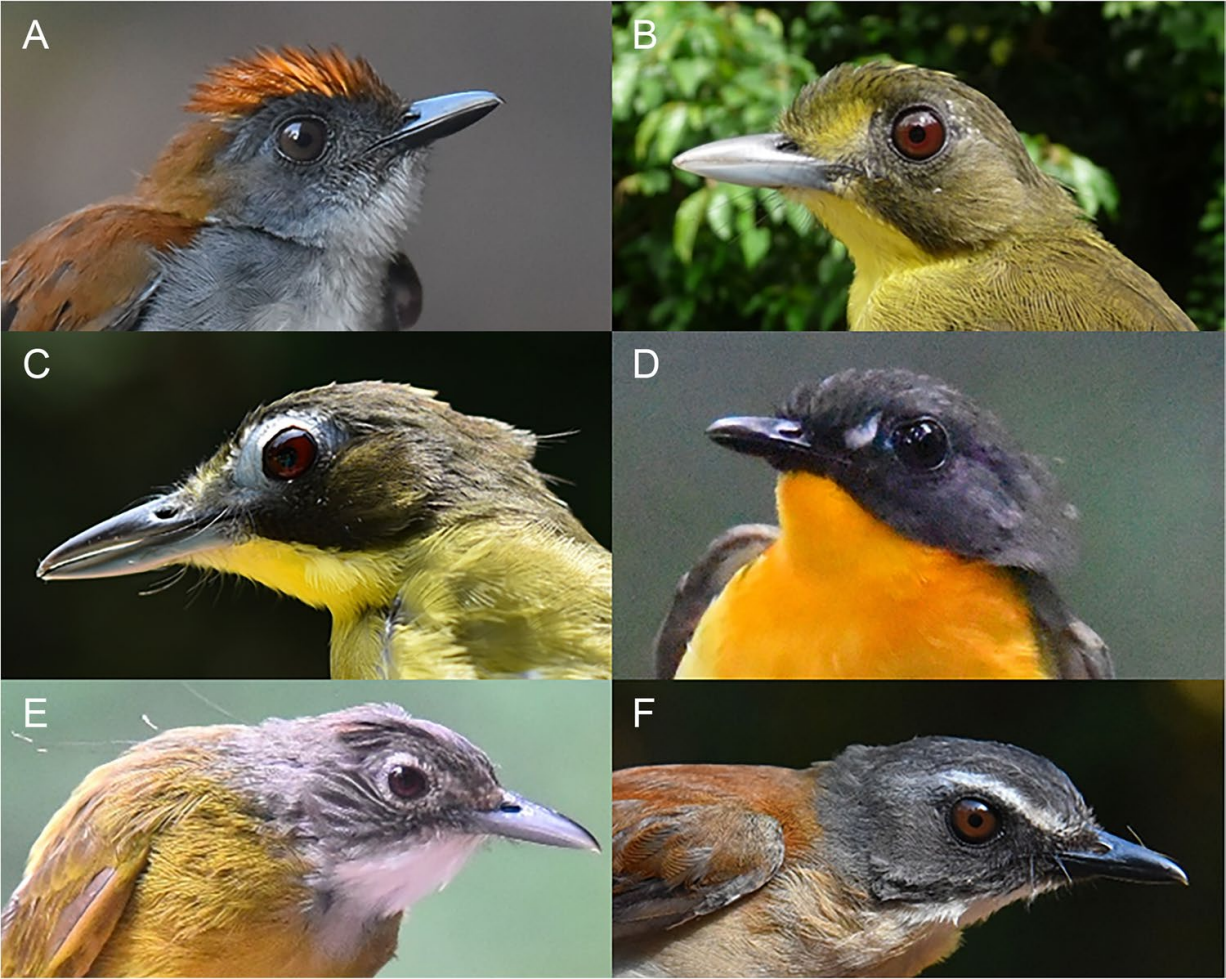
Bird species with highest driver ant association indices based on data from lowland forest, Korup National Park, Cameroon (left to right, from top to bottom: fire-crested alethe *Alethe castanea* (**A**), lesser bristlebill *Bleda notatus* (**B**), red-tailed bristlebill *Bleda syndactyla* (**C**), forest robin *Stiphrornis erythrothorax* (**D**), red-tailed bristlebill *Criniger calurus  *(**E**), brown-chested alethe *Chamaetylas poliocephala* (**F**)

In the interspecific comparison, there was a negative relationship between the proportion of raids attended (Table 2) and density (including army ant-followers observed and those not-observed feeding on arthropods flushed), with birds of high density attending flocks less frequently compared to birds of low density (Fig. 1, *p* < 0.1). According to our model, data points for the following species were higher than average and outside the 95% confidence interval (assuming a higher association with army ants than expected by their density): brown-chested alethe (B-A), white-tailed ant thrush (WAT), and red-tailed greenbul (R-G) had data points being only slightly outside the 95% CI, whereas those of red-tailed (R-B) and lesser bristlebill (LsB), forest robin (FrR), and fire-crested alethe were more than 25% higher than the upper 95% CI (Fig. 1). The fire-crested alethe was the most specialized ant-following bird (Figs. 1, 2, and 3). When plotting true raid attendants only (species observed feeding at ant swarms), density has a significant (*p* < 0.01, black solid line) positive effect on raid attendance by number of individuals (Supplementary Material C, Fig. 9).

### Morphological and plumage traits

**Fig. 4:**
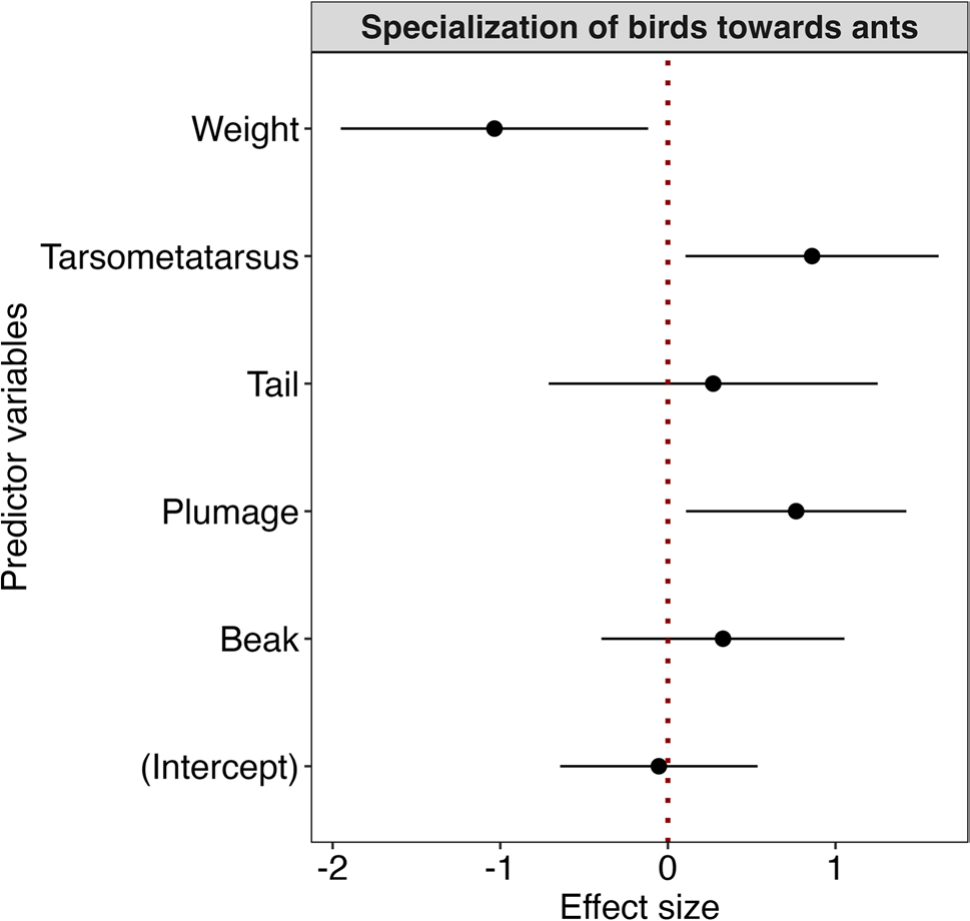
Effect sizes of the predictor variables (bird species’ traits) fitted for modeling the proportion of Afrotropical bird species at ant swarms using generalized linear modeling. Data from lowland forest, Korup National Park, Cameroon

Our analysis provided some evidence that species’ morphology and plumage traits predict its specialization towards ant raids: in a multi-model inference framework, we found that three variables, i.e., metatarsus length, plumage traits, and weight, were the best predictors of each species’ proportions at raids (i.e., in the best-fitting model, AIC_weight_ = 0.31; Table 3). In our model, birds attending swarm raids of army ants were predicted to have a large metatarsus length, to be relatively small and to show plumage traits related to aggression behavior. The second best-fitting model additionally included a positive effect of the *beak size* variable (AIC_weight_ = 0.17; Table 3). Confidence intervals did not overlap zero for the variables weight (negative values), plumage traits, and metatarsus length (positive) (Fig. 4).

**Table 3:** Parameters of the best-fitting generalized linear model to explain attendance rates of birds at ant swarms in lowland forest, Korup National Park, Cameroon

Model	Intercept	Parameters					LogLik	∆AICc	AICc weight
		beak	MtTars	plsign	tail	weight			
m23	− 0.29		1.13 (0.42)	1.19 (0.36)		− 0.92 (0.41)	− 34.93	0.00	0.33
m24	− 0.29	0.71	1.06 (0.40)	1.59 (0.43)		− 1.17 (0.43)	− 33.40	1.12	0.19
m5	− 0.29			1.11 (0.41)			− 39.08	1.53	0.15
m7	− 0.29		0.66 (0.41)	1.08 (0.35)			− 37.56	1.64	0.14
m31	− 0.29		1.00 (0.41)	1.26 (0.35)	0.71 (0.52)	− 1.38 (0.53)	− 33.71	1.79	0.13
M6	− 0.29	0.47 (0.48)		1.36 (0.48)			− 38.53	3.60	0.05

The values in the parenthesis show the standard error of the beta coefficients (*β*). AICc indicates the Akaike information criterion corrected for small sample size. LogLik is the logarithmic likelihood ratio. ∆AICc is the delta AICc

Abbreviations: *MtTars* mean adult tarsometatarsus length in mm, *plsign* plumage signals, *beak* mean adult culmen length in mm, *tail* mean adult tail length in mm, *weight* mean adult weight in g

## Discussion

In the Neotropics, the number of bird individuals following swarming ants can vary widely even on a small regional scale (Willis and Oniki 1978). In our study in Central African lowland rainforest, flock sizes ranged from 5 to 11 individuals (mean = 5.34 ± 2.68 SD) with a flock species richness of four to ten species (mean = 6.89 ± 2.1SD). We encountered ant-following birds at ant swarms only on 9 of 22 occasions. This encounter rate of ant raids as well as the size of flocks was perceived to be much lower compared to other years (MW mistnetted yearly in the study area since 2010). Compared to East Africa, species richness of aggregations was comparable (Kenya 6.16 ± 3.54, mean ± SD), but aggregation sizes were larger on average (mean = 11.81 ± 9.64), and aggregations varied more compared to Kenya (Peters et al. 2008).

With the exception of two species which do not occur in Kakamega, also, the species composition of the flocks was similar between the two studies, and there was also strong overlap in patterns of specialization in regard to ant following (Peters et al. 2008). Interestingly, the most specialized ant-following species in Kakamega Forest (Peters et al. 2008) were also observed in our study in Cameroon’s Korup National Park and did not show overly strong differences with respect to their estimated strength of specialization as ant-followers. The ranks of the two species showing the highest specialization in the Kakamega study, white-tailed ant thrush and brown-chested alethe, were taken in our study by fire-crested alethe and lesser bristlebill which both do not occur in Kakamega: these two species also show strong dominant behavior at ant raids. In our study, they were followed by forest robin and red-tailed bristlebill, and brown-chested alethe and white-tailed ant thrush, ranking next in regard to specialization. The red-tailed bristlebill was found to be strongly specialized in the Kenyan study as well (Peters et al. 2008). The bird composition of our study site is typical of a lowland rainforest avifauna, while Kakamega Forest lies at the Western Plateau of Kenya, at a height of 1500 to 1600 m a.s.l., where Guineo-Congolian species occur side by side with montane species (Serle 1979; Wandago 2003; Peltorinne 2004).

Overall, the small numbers of encountered raids observed by us may have been a consequence of the high temperatures occurring during the dry season 2015/2016 during which our study was conducted. The high temperatures were associated with the strong El Niño Southern Oscillations recorded in the same period (Goldiner 2015; UNOCHA 2016). Compared to *Dorylus mayri*, *Dorylus sjoestedti* ants are more active during dry seasons, and accordingly where also more commonly encountered during fieldwork (ratio 18:3), *D. mayri* seems to prefer cool and humid conditions (Raigner and van Boven 1955; Deblauwe and Dekoninck 2007). During our study, both species’ activity might have been reduced by the unusually dry conditions, similar to ant species in the Neotropics (Schneirla 1949; Delsinne et al. 2008), resulting in a lower number of total raids in both species.

The knowledge that has so far been assembled on the ant-following behavior of birds on the African continent is mostly based on species accounts in handbooks and field guides. The white-tailed ant thrush, for example, is described as a very consistent raid attendant showing dominance towards both brown-chested and fire-crested alethe (Brosset and Erard 1986). In the bird flocks we were able to observe thoroughly, the white-tailed ant thrush was a rarer guest and never showed a sign of aggressiveness towards any other bird individual. Instead, fire-crested alethe appeared to be an extremely dominant species itself: it did not only demonstrate aggressive behavior towards conspecifics (as mentioned in Willis 1986 and Brosset and Erard 1986), but we also witnessed individuals of the species defending their central positions at the front of an ant column against several lesser bristlebills and individuals of brown-chested alethe. As fire-crested alethe and lesser bristlebill were the most present and abundant species in our raids observed, it often came to “territorial” quarrels for the best positions in foraging at an ant raid. In his article on West African thrushes as safari ant-followers, Willis (1986) also describes the fire-crested alethe as raid attendant “following them more than half the time” and that it can be seen visiting inactive ant colonies in the early morning while looking for ant raids. Likewise, Brosset and Erard (1986) refer to the profound knowledge gained by fire-crested alethes on the activities of an ant colony. This behavior seems quite comparable to the ant colony or bivouac checking performed by opportunistic ant-followers in South America (Swartz 2001; O’Donnell et al. 2010; Chaves-Campos 2011).

Interestingly, the blue-headed wood dove has not yet been reported as possible ant-follower (Waltert et al. 2005; Borrow and Demey 2001, 2014), but one individual has been detected foraging among a column of *D. sjoestedti* ants crossing a trail, where it was uninterruptedly picking small items among the ants. Although this wood dove species is accounted for as belonging to the guild of granivorous birds, insect and fruit predation occurs as well (Waltert et al. 2005; Baptista et al. 2017).

Although African tropical rainforests are said to hold few specialized ant-followers, the results obtained in this study and numbers from Peters et al. (2008) reveal that they might not be far behind in numbers of specialists compared to South American forests. Four to seven specialized ant-followers were identified for the Cameroonian lowland rainforests in this study and five specialist species in Kakamega Forest, Eastern Africa (Peters et al. 2008), compared to six identified specialists in montane forests of Costa Rica (O’Donnell et al. 2010), all categorized in the same way by regression of raid attendance on point-count densities. Additionally, two more species showed specialized behavior (bivouac checking) in the Costa Rican study.

### Plumage traits associated with ant-following behavior

Our findings further indicate that certain plumage traits were significantly associated with the specialization of birds towards ants. All species with a specialization index larger than 0 do have some kind of plumage ornaments. The most specialized ant-follower, the fire-crested alethe, has an erectable crown, which contrasts the gray facial color (Fig. 3). Other species with a high index such as lesser and red-tailed bristlebill or forest robin do have spots of bright plumage near the eye, and/or a bare ring around the eye (Fig. 3). This is similar also in obligate Neotropical antbirds Thamnophilidae (e.g., *Myrmeciza fortis*, *Phlegopsis nigromaculata*). Red-tailed greenbul, white-tailed ant thrush, and brown-chested alethe do also show plumage traits to a variable extent and also have a specialization index above zero. While brown-chested alethe shows a prominent white superciliary streak; the ant thrush and the red-tailed greenbul have conspicuous tail coloration/markings.

Our hypothesis was that successful ant following should be related to some form of dominance or aggression towards conspecifics or other species competing for food around swarms. While we did not directly measure aggression or dominance in our study, there is evidence from species accounts in handbooks that the most strongly ant associated and signaling species, e.g., fire-crested alethe and red-tailed bristlebill, are fiercely defending this important food resource. Further, it is well-known that in many passerine species, aggression and dominance correlates with conspicuously colored plumage patterns which serve as signals in antagonistic encounters (Chaine et al. 2013; Galvan 2010; Jaboński and Matyjasiak 2002; Jawor and Breitwisch 2003; Leitão et al. 2019; Swaddle and Witter 1995; van Dongen and Mulder 2007; Yasukawa et al. 2009).

### Other morphological features

Our study suggests that terrestrial foraging and the associated strength and length of metatarsi are favorable predispositions or useful adaptations (or both) when specializing on ant following. Especially thrushes and terrestrial bulbuls are predisposed in this way and seem to benefit from ant following in largely undisturbed or only moderately disturbed forests. Most ant raid-attending birds are known to be also terrestrial ground foragers (Waltert et al. 2005), and that was also the case in our study. We expected these to show morphological characteristics related to ground dwelling. One of these traits is especially strong and long *metatarsi*, being a feature of terrestrial foragers (Zeffer and Lindhe Norberg 2003; Provini and Höfling 2020). Indeed, this metric turned out to be relevant in our models, since all thrushes, as well as the bristlebills, and the red-tailed greenbul indeed have long and strong legs, exemplifying terrestrial habits. In our second best model, also *beak size* was included. This is not surprising since strongest beaks occur in three of the most specialized (to ants) species, namely red-tailed bristlebill, little bristlebill, and red-tailed greenbul. We were expecting also *weight* to increase with increasing with specialization to ants, i.e., larger species being more specialized than smaller species. However, this was not the case in our dataset, since one of the more specialized species was a small species (the forest robin) and one of the least specialized species was very large (blue-headed wood dove). The specialization index of the forest robin may indicate that also small birds may be strongly associated with ant swarms, as has also been found in the Neotropics. There may also be good opportunities for small species to complement their diet with the many small prey items disturbed by ants. Since *Dorylus* ants are sedentary (not bivouacking), and repeatedly scan the forest floor for prey, in the surroundings of their nests, large prey items could become relatively rare compared to smaller sized arthropod prey, which are suitable as food for smaller thrushes.

## Conclusion

Our study suggests that the ant-following phenomenon of the forests of the Guinean-Congolian region is fairly similar to that of East Africa (e.g., Peters et al. 2008). In addition, the study supports the idea that successful army ant following may be related to a predisposition towards terrestrial foraging and that certain plumage signals support dominance behavior around ant swarms. These adaptations make valuable food resources of the forest floor better available to birds. The morphological traits and behavioral strategies that ant-followers use deserve further investigations, still, both in Africa as well as the Neotropics.

### Supplementary Information

Below is the link to the electronic supplementary material.

Supplementary file1 (DOCX 270 KB)
