# Supplementary material for: Ant-following behavior is correlated with plumage traits in African understory birds
Source: Naturwissenschaften. 2024 Jul 30;111(4):41. doi: 10.1007/s00114-024-01927-3 (PMC11289350; doi:10.1007/s00114-024-01927-3)
Supplement: Supplementary file 1 — Supplementary file1 (DOCX 270 KB) [file 114_2024_1927_MOESM1_ESM.docx]

Supplementary Material A

In order to assess whether we identified most/all species which attend ant swarms in our study area, smoothed species accumulation curves were calculated, using the number of species [mean ± SD] in relation to study effort (number of flocks at raids observed) to estimate the increase in species-richness. Four estimators of species richness were applied: Abundance Coverage-based Estimator (ACE), Chao1 richness estimator for abundance data, Chao2 richness estimator for replicated incidence data and first-order Jacknife richness estimator (Jack1) (Colwell, 2013). These estimators include species that were not detected in the samples and therefore provide an estimation of total species richness.

Species accumulation curves by number of species observed following army ants during field work in Korup National Park, Cameroon. Extrapolating the 21 species identified in nine flocks, more than 30 species are to be expected with an increase of flocks encountered according to Abundance Coverage-based Estimator (ACE). Chao 1 and Chao 2 curves represent the lower bounds of predicted species richness, but still lie higher than measured richness, which reaches 70 % of Chao 1 and 72 % of Chao 2 at the maximum (n=9). Labels: S(est): estimated number of species represented by the sample; ACE: Abundance-Coverage-based Estimator; Chao 1: richness estimator for abundance data; Chao 2: richness estimator for replicated incidence data; Jack 1: first order Jackknife for abundance data.

Colwell, R. K. (2013): EstimateS 9.1.0 User’s Guide. University of Connecticut, CT. <http://viceroy.eeb.uconn.edu/estimates/EstimateSPages/EstSUsersGuide/EstimateSUsersGuide.htm>; [26.01.2017; 10:08])

Supplementary Material B

## **Bird densities from point-count data**

Density estimates from a Distance analysis for all species observed in proximity and feeding at army ant raids. The study effort was 168 point counts with a duration of 10 minutes each. Abbreviations: #obs: Number of observations; Trunc.: Truncation at maximum distance; EDR: effective detection radius; D: Estimated density of individuals; DS: Estimated density of clusters; P: Detection probability.

| **Species** | **#obs** | **Trunc.** [m] | **EDR** [m] | **D** [Ind/km²] | **DS** [CS/km²] | **P** |
| --- | --- | --- | --- | --- | --- | --- |
| Red-tailed Bristlebill | 40 | 43 | 29.21 | 97.002 | 88.807 | 0.46 |
| Lesser Bristlebill | 25 | 30 | 15.29 | 202.622 | 202.622 | 0.26 |
| Red-tailed Greenbul | 35 | 30 | 16.01 | 289.796 | 258.869 | 0.28 |
| Eastern-bearded Greenbul | 51 | 50 | 28.74 | 122.243 | 116.975 | 0.33 |
| Yellow-whiskered Greenbul | 38 | 28 | 20.72 | 200.573 | 167.784 | 0.55 |
| Little Greenbul | 356 | 42 | 24.74 | 1251.031 | 1102.236 | 0.35 |
| Forest Robin | 42 | 32 | 28.62 | 97.166 | 97.166 | 0.80 |
| Fire-crested Alethe | 79 | 40 | 28.41 | 185.432 | 185.432 | 0.50 |
| Brown-chested Alethe | 47 | 40 | 30.84 | 93.633 | 93.633 | 0.59 |
| White-tailed Ant Thrush | 20 | 30 | 15.67 | 154.731 | 154.731 | 0.27 |
| Rufous Flycatcher Thrush | 36 | 40 | 19.00 | 197.887 | 188.906 | 0.23 |
| Pale-breasted Illadopsis | 13 | 32 | 21.13 | 60.441 | 55.146 | 0.44 |
| Brown Illadopsis | 46 | 35 | 23.05 | 157.205 | 164.007 | 0.43 |
| Grey Longbill | 67 | 50 | 32.27 | 119.264 | 118.562 | 0.43 |
| Yellow Longbill | 8 | 31 | 31.00 | 15.776 | 15.776 | 1.00 |
| Red-bellied Paradise Flycatcher | 16 | 25 | 16.67 | 109.082 | 109.082 | 0.44 |
| Green Hylia | 42 | 27 | 18.26 | 238.701 | 238.701 | 0.46 |
| Olive Sunbird | 159 | 50 | 12.68 | 1902.712 | 1873.912 | 0.06 |
| Yellow-bellied Wattleeye | 2 | 25 | 25.00 | 6.064 | 6.064 | 1.00 |
| Blue-headed Wood Dove | 54 | 100 | 54.22 | 34.789 | 34.789 | 0.29 |

Supplementary Material C

A second specialization-index was calculated for each species as the proportion of individuals contributed to all flocks at army ant raids divided by the total number of bird individuals at raids. These results were very similar to those being based on the proportion of raids attended by each species out of all raids. The model applied using this measure of attending-rate was able to explain 99% of the individual-based data with density of species in the study area as explanatory variable in a Likelihood Ratio Test. There was also a negative correlation between density and raid attending rate, meaning that birds of high density attended flocks in lower number of individuals compared to birds of low density (Fig. 7). Again, this pattern was strongly influenced by values of Little Greenbul and Olive Sunbird, which were the species with highest densities in the study area, but contributed only relatively all numbers of individuals at raids (Tab.2).


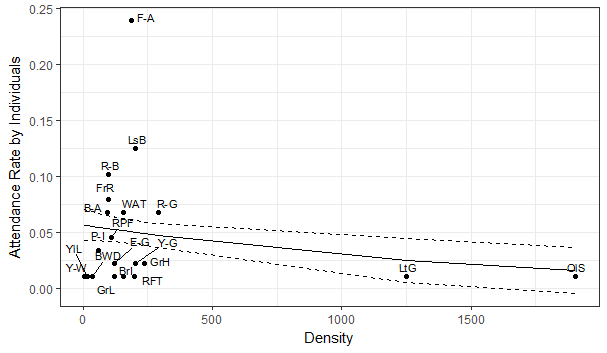


Figure 7: GLM plotting of raid-attending rate by number of individuals per species from all individuals present (ra-II, Tab.2) against species density in the study site from point counts. Species above the 95% CI (upper dashed line) are identified as specialized ant-followers. Density has a negative significant effect (P<0.01, black line) on raid attendance r-II, strongly influenced by the values of Little Greenbul (LtG) and Olive Sunbird (OlS). For species labels see Fig. 3).

Species above the 95% CI of the modelled correlation of density and attendance rate are those with possible specialization for ant-following. By application of this model seven species can be identified with different degrees of specialized behavior for ant-following (Fig. 8).


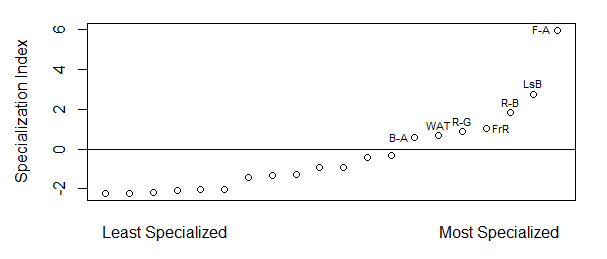


Figure 8: Ant-following indices by number of individuals per species in ordered sequence with specialized ant-followers labelled (see also Fig. 2).

Brown-chested Alethe, White-tailed Ant Thrush and Red-tailed Greenbul are slightly outside the 95% CI which would indicate a low specialization; Red-tailed and Lesser Bristlebill as moderate specialists are located more than 0.05 and 0.1 above and the species with the highest number of individuals in all raids observed, was Fire-crested Alethe, once again underlining its high level of specialization for ant-following.

Excluding non-feeding ant-followers from the model, density was able to predict the variation in raid attendance by low significance in a Likelihood Ratio Test, explaining the number of individuals taking part as raid attendants in dependence of species’ density in the study area (Fig. 11). Three species can finally be identified as moderate to high specialized ant-followers, namely Red-tailed and Lesser Bristlebill, and Fire-crested Alethe.


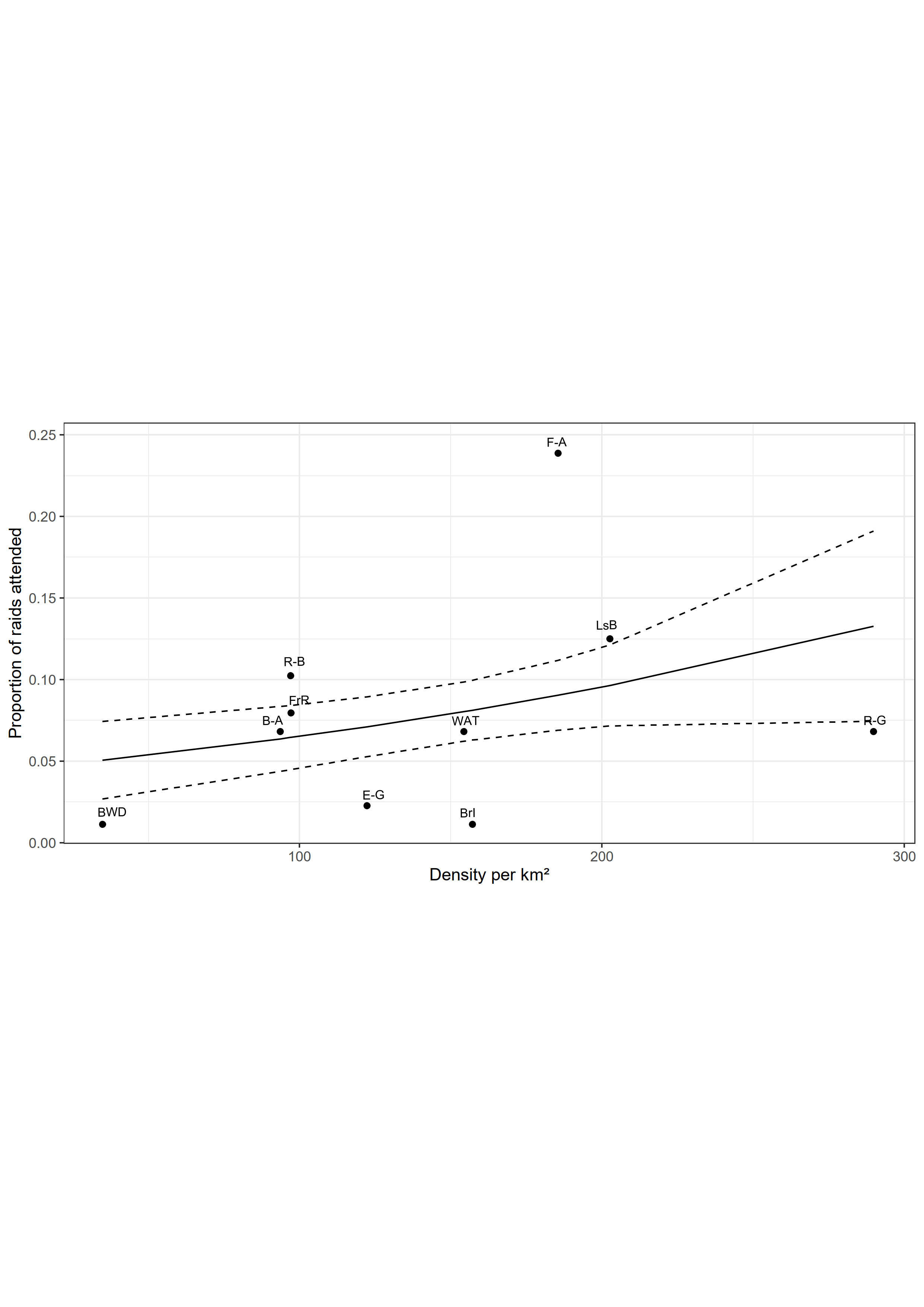


Figure 9: When plotting true raid attendants only, density has a significant (P<0.01, black solid line) positive effect on raid attendance by number of individuals. Species above the 95% CI (upper dashed line) are identified as specialist species, namely Red-tailed Bristlebill (R-B), Lesser Bristlebill (LsB) and – as most specialized ant-follower in KNP – Fire-crested Alethe (F-A) (other species labels, see Fig. 4).
